# Exploring gender differences among couples with unexplained recurrent pregnancy loss regarding preferences for supportive care

**DOI:** 10.1186/s12884-021-04277-4

**Published:** 2021-11-30

**Authors:** N. A. du Fossé, E. E. L. O. Lashley, T. T. Treurniet, J. M. M. van Lith, S. le Cessie, H. Boosman, M. L. P. van der Hoorn

**Affiliations:** 1 Department of Gynecology and Obstetrics, Leiden University Medical Center, Albinusdreef 2, Leiden, 2333 ZA the Netherlands; 2 Department of Clinical Epidemiology, Leiden University Medical Center, Leiden, 2333 ZA the Netherlands; 3 Department of Biomedical Data Sciences, Leiden University Medical Center, Leiden, 2333 ZA the Netherlands; 4 Department of Quality and Patient Safety, Leiden University Medical Center, Leiden, 2333 ZA the Netherlands

**Keywords:** Recurrent pregnancy loss, Recurrent miscarriage, Supportive care, Male perspective

## Abstract

**Background:**

International guidelines recommend to offer supportive care during a next pregnancy to couples affected by recurrent pregnancy loss (RPL). In previous research, several options for supportive care have been identified and women’s preferences have been quantified. Although it is known that RPL impacts the mental health of both partners, male preferences for supportive care have hardly been explored.

**Methods:**

A cross-sectional study was conducted in couples who visited a specialized RPL clinic in the Netherlands between November 2018 and December 2019. Both members of the couples received a questionnaire that quantified their preferences for supportive care in a next pregnancy and they were asked to complete this independently from each other. Preferences for each supportive care option were analysed on a group level (by gender) and on a couple level, by comparing preferences of both partners.

**Results:**

Ninety-two questionnaires (completed by 46 couples) were analysed. The overall need for supportive care indicated on a scale from 1 to 10 was 6.8 for men and 7.9 for women (*P* = 0.002). Both genders preferred to regularly see the same doctor with knowledge of their obstetric history, to make a plan for the first trimester and to have frequent ultrasound examinations. A lower proportion of men preferred a doctor that shows understanding (80% of men vs. 100% of women, *P* = 0.004) and a doctor that informs on wellbeing (72% vs. 100%, *P* = ≤0.000). Fewer men preferred support from friends (48% vs. 74%, *P* = 0.017). Thirty-seven percent of men requested more involvement of the male partner at the outpatient clinic, compared to 70% of women (*P* = 0.007). In 28% of couples, partners had opposing preferences regarding peer support.

**Conclusions:**

While both women and men affected by RPL are in need of supportive care, their preferences may differ. Current supportive care services may not entirely address the needs of men. Health care professionals should focus on both partners and development of novel supportive care programs with specific attention for men should be considered.

**Supplementary Information:**

The online version contains supplementary material available at 10.1186/s12884-021-04277-4.

## Background

Recurrent pregnancy loss (RPL) is a frustrating condition for both patients and care providers. This condition, defined as the loss of two or more pregnancies before the fetus reaches viability, is estimated to affect 1-3% of all couples of reproductive age [1–3]. Multiple risk factors have been identified, but despite extensive diagnostic investigations, RPL remains unexplained in the 60-70% of cases [4]. For these couples, there is currently no evidence-based medical treatment option. As pregnancy losses are generally experienced as significant negative life events, RPL may have serious psychological impact. A recent study reported that both women and men affected by RPL show high risks for developing depression and anxiety, while they often use different coping strategies [5].

It is recommended by current international guidelines to offer supportive care programs for couples with RPL [6]. Some studies even suggested that supportive care during early pregnancy may have a beneficial effect on pregnancy outcome, although this evidence is limited [7–10]. Moreover, professional support and compassionate care are highly valued by couples with RPL [11]. Musters et al. elucidated what is actually perceived as supportive care for RPL and evaluated women’s preferences for 20 supportive care options during a next pregnancy [12, 13]. They showed that women with RPL preferred to see the same doctor during their consultations who is specialized in RPL, takes them seriously, listens, shows understanding and enquires about emotional needs. The women wanted to make a plan with their doctor for the first trimester of a new pregnancy and they preferred frequent ultrasound examinations during this period. Furthermore, they indicated a need for psychological after-care in case of a new miscarriage. Notably, male partners’ preferences and their need for supportive care were not addressed in this study.

As shown by a systematic review [14] that evaluated 27 studies on patient-centred early pregnancy care, male partners were not involved in most prior studies in this research field. The male perspective was examined in only three of the included studies and the authors considered involvement of the partner as an improvement target. Identifying male preferences for supportive care in RPL is relevant, not only because it has been shown that men do also suffer from RPL, but also because tailored supportive care programs may assist the male partner during a new pregnancy. The significance of this has been underscored by several studies showing that the male role in pregnancy is of great impact on maternal health behaviour and pregnancy outcome [15–17].

The aim of the current study was to quantify preferences for supportive care of both men and women affected by RPL. Previously identified supportive care options for RPL [12, 13] were used as a framework for this study and both members of participating couples were independently questioned, allowing us to compare preferences between genders but also to analyse potential discrepant preferences within couples.

## Methods

### Participants

This cross-sectional study was conducted in couples that visited the specialized RPL outpatient clinic of the Leiden University Medical Center in the Netherlands between November 2018 and December 2019. Participating couples had at least two pregnancy losses (following the definition of the ESHRE guideline for RPL [1]) and had to be fluent in Dutch or English. The study protocol was approved by the Medical Research Ethics Committee of the Leiden University Medical Center (reference number N19.101). All participants provided written consent to take part in the study.

### Procedures at the RPL outpatient clinic

When couples visit the RPL clinic for the first time, they have an intake consultation with a gynaecologist or fertility doctor. The team comprises four physicians, all specialized in RPL. All physicians adhere to the same protocol and provide similar care. New patients are discussed in the team after their first consultation. Besides obtainment of detailed obstetric history and extensive history of both partners, couples receive information about known risk factors for RPL, advices on lifestyle changes, options for diagnostic testing, potential therapeutic options, chances for future pregnancy outcome and ongoing studies.

Besides the medical approach, attention is paid to the psychological impact of RPL and consultation with a medical social worker is offered. A referral can be made immediately, or the couple can make an appointment at a later time if desired (it is estimated that 10% of all couples opt for a consultation with the medical social worker). In case of a next pregnancy, couples are offered monitoring at the RPL outpatient clinic in the first 12 weeks of the pregnancy. Ultrasound examination in the first trimester is offered, the frequency depending on the couple’s preference. In addition, it is emphasized that the affiliated obstetric clinic of the Leiden University Medical Center is available ‘twenty-four seven’ and can be reached in case of any symptoms or distress. In case of an ongoing pregnancy beyond 12 weeks, the couple will be referred for further regular monitoring of the pregnancy to either an obstetrical outpatient clinic or a midwifery practice (depending on medical indication and individual situation). In case of another pregnancy loss, the doctor will re-evaluate their individual plan at the follow-up consult at the RPL outpatient clinic.

### Data collection

After the couples had attended the intake consultation, they received printed questionnaires, which were completed at home. The questionnaires were returned by post or during a next consultation. The questionnaire consisted of two parts: general demographic questions and preferences for supportive care. The second part of the questionnaire was based on supportive care options in three domains as identified by Musters et al. [12, 13]: 1: Medical supportive care (for example: ultrasound examination during early pregnancy, medical information and advices); 2: Soft-skills (for example: communication skills of the doctor) and 3: Other types of supportive care (for example: support from friends, family and peers, relaxation exercises, alternative therapies).

Two versions of the questionnaire were used, intended for either women or men. Given the purpose of the study, the couples were asked to complete the questionnaires independently, without discussion between both partners. The questionnaires were available in Dutch and English language (the English version is included as Supplementary material). Preferences and need for supportive care were quantified using 5-point Likert scale items ranging from total disagreement to total agreement and a rating scale question (grade 1-10). The estimated completion time for the questionnaire was maximum 15 min. The questionnaires were developed and pilot tested by two gynaecologists (specialized in RPL), two fertility doctors (specialized in RPL), a psychologist, a PhD candidate (specialized in RPL) and two patients with RPL. No major adjustments were made after pilot testing.

### Statistical analyses

Descriptive data are presented in numbers and percentages. The 5-point Likert scale items for supportive care options were recoded: 1 and 2 represent the non-preference group, 3 the neutral group, and 4 and 5 the preference group (similar to Musters et al. [13]). Scale reliability was assessed with Cronbach’s alpha. To prevent multiple hypothesis testing, statistical tests were not executed for the complete panel of supportive care options but restricted to predefined selected entities: whenever a supportive care option was preferred by either ≥60% of women, ≥60% of men, or both, this option was considered as potentially relevant for clinical practice and thus examined in further detail. This was done by comparing the preference rates for these selected supportive care options between women and men. To account for the statistical dependence of data derived from two partners of a couple, McNemar tests for paired data were used. The mean overall need for supportive care expressed on a scale from 1 to 10 is presented with standard deviation (SD) and compared between women and men with a paired samples T-test. Two-sided *P*-values < 0.05 were considered statistically significant. Intra-couple discrepancy was defined as one of the two partners having no need (1 or 2) for a certain supportive care option and the other partner having a preference (4 or 5) for this supportive care option. The level of intra-couple discrepancy for each supportive care option was calculated as the percentage of all couples that met this definition. Analyses were performed in R studio version 1.3.9.50 (R Foundation for Statistical Computing, Vienna, Austria).

### Sample size calculation

On the basis of the null hypothesis that an equal percentage of women and men would prefer a supportive care option, a sample size of 44 couples would be required for an 80% power at a two-sided alpha of 0.05 to detect a difference in preference rate of 30% between women and men, which we considered as a clinically relevant difference. The sample size was calculated with R studio package ‘SampleSizeMcNemar’.

## Results

Between November 2018 and December 2019, 50 women and 46 men completed the questionnaire. Four questionnaires were excluded from the analyses as only the female partner returned the questionnaire. All couples were heterosexual. The majority of women and men (85% both) were born in the Netherlands. The median number of pregnancy losses at the time the RPL outpatient clinic was visited for the first time was 2 (range 2-6). No underlying condition for RPL was found in 70% of the couples. More baseline characteristics of the couples are shown in Table 1.

**Table 1:** Baseline characteristics of couples with RPL

**Baseline characteristics of couples with RPL** *n* = 46		
Referral by *n* (%)		
Physician of same hospital		18 (39)
General practitioner		10 (22)
Midwife		5 (11)
Secondary hospital		13 (28)
Reproductive information		
Number of pregnancy losses (median)		2 (range 2–6)
Couples with child together *n* (%)		21 (46)
Fertility treatment *n* (%)		
IVF		2 (4)
IUI only		4 (9)
None		40 (87)
Pregnant during intake consultation *n* (%)		5 (11)
RPL diagnosis *n* (%)		
Unexplained		32 (70)
Thyroid autoimmunity		6 (13)
Uterine anomaly		4 (9)
Unknown (no diagnostic work-up)		2 (4)
Antiphospholipid syndrome		1 (2)
Parental chromosomal translocation		1 (2)
	**Women** *n* = 46	**Men** *n =* 46
Age (mean, (SD))	34 (4.40)	37 (5.58)
Education level		
Low^a^	1 (2)	3 (7)
Moderate^b^	13 (28)	14 (30)
High^c^	32 (70)	29 (63)

*IVF* In vitro fertilization, *IUI* Intrauterine insemination, *RPL* Recurrent pregnancy loss

^a^Primary school/intermediate vocational education

^b^Higher general secondary education/pre-university secondary education

^c^Higher vocational education/university

### Preferences for supportive care in a next pregnancy

**Fig. 1:**
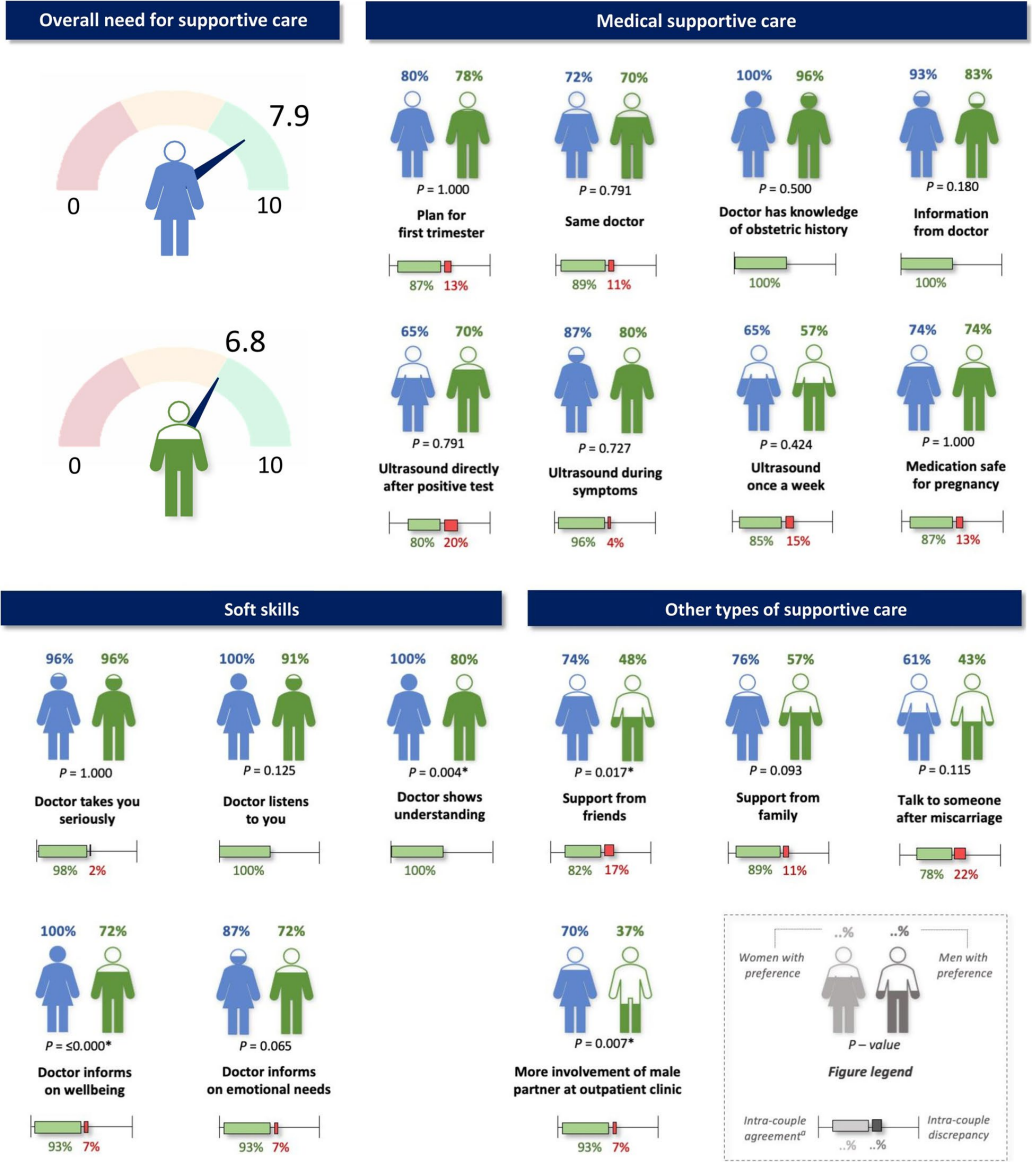
Overall need for supportive care of women and men affected by RPL and options for supportive care in a next pregnancy preferred by the majority (≥60%) of women and/or men. Overall need for supportive care was measured on a scale from 1 to 10, mean values for both genders are shown. For each supportive care option, preference rates for women and men with *P*-values and levels of intra-couple discrepancy (as defined in the Statistical analysis section) are shown. Further explanation is shown in grey text in the bottom right corner. ^a^ Intra-couple agreement: both partners indicated a preference or a non-preference, or one partner responded neutral. Asterisks (*) indicate *P*-values < 0.05

**Fig. 2:**
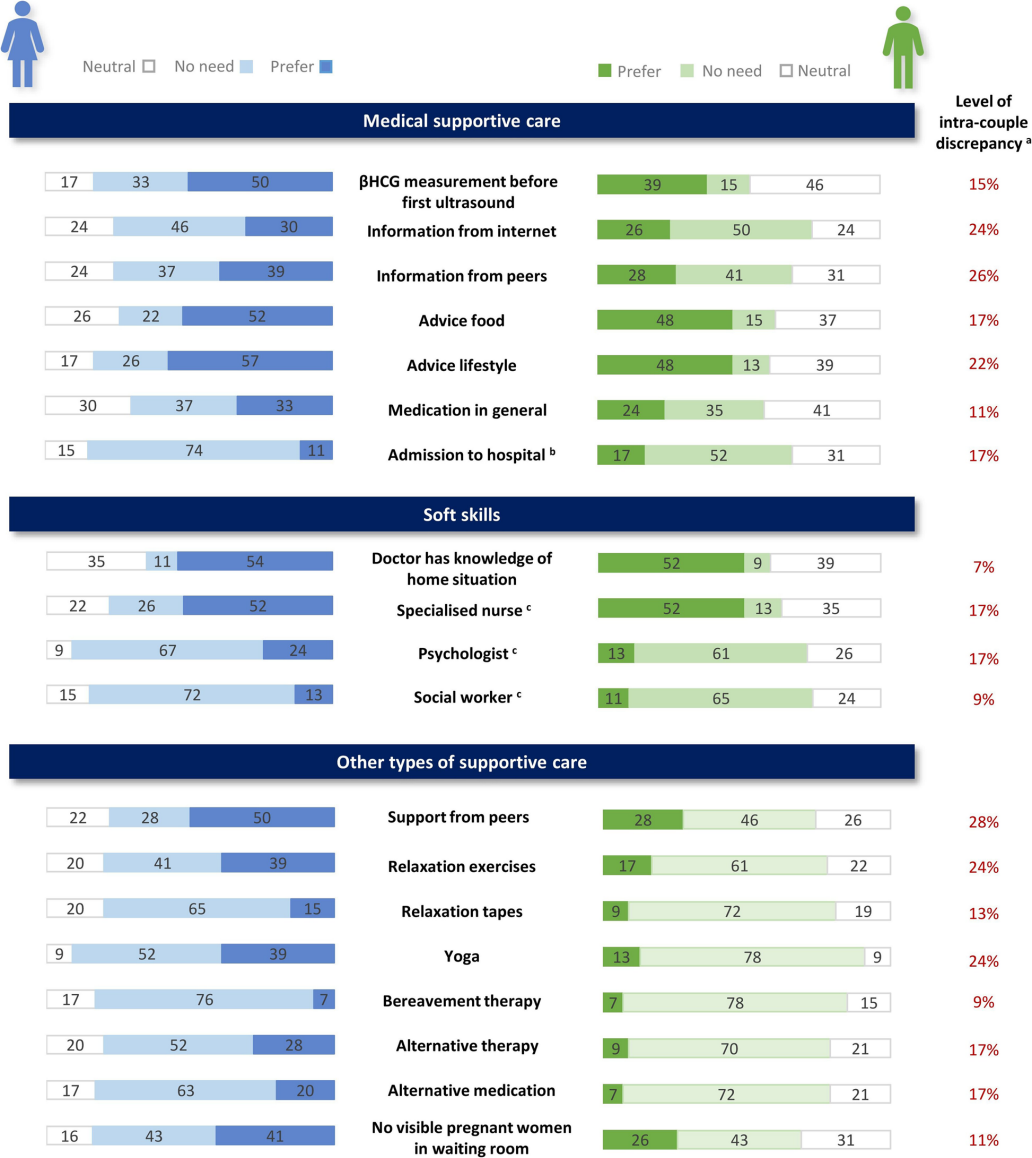
Options for supportive care in a next pregnancy preferred by < 60% of women and men affected by RPL. ^a^ Level of intra-couple discrepancy: % of couples with opposing opinions (i.e. one partner indicated a preference and the other partner indicated no need), as described in the Statistical analysis. ^b^ Admission to hospital at same gestational age as earlier miscarriages occurred. ^c^ Counselling from mentioned specialist

The mean need for supportive care expressed on a scale from 1 to 10 was 6.8 (SD 1.68) for men and 7.9 (SD 1.65) for women (*P* = 0.002). Overall, Cronbach’s alpha was 0.82 (0.80 for the subgroup of women and 0.82 for the subgroup of men), indicating good reliability of the Likert scales. Seventeen options for supportive care in a next pregnancy were preferred by either the majority (≥60%) of women and/or men. Preference rates and levels of intra-couple discrepancy for these specific options are shown in Fig. 1, including *P-*values for the differences in preference rates between women and men. In Supplementary Table 1, also the percentages of women and men that scored neutral for these options are shown. An overview of the other supportive care options, being preferred by < 60% of women and men, is shown in Fig. 2.

#### Domain 1: medical supportive care

The majority of both women and men preferred making a plan for the first trimester, seeing the same doctor during different consultations who has knowledge of their obstetric history, an ultrasound examination directly after a positive test, once a week during the first trimester and during symptoms and medication for RPL that is proven safe for pregnancy. Medication that is not proven safe during pregnancy (i.e. experimental medication for RPL without fully known effects and safety) was preferred by 33% of women and 24% of men. Information derived from a doctor was preferred over information derived from the internet or information derived from peers. On group level, there were no significant differences between genders for all of the above options. The levels of intra-couple discrepancy were highest for the options information from peers (26%), information from the internet (24%) and advice regarding lifestyle (22%).

#### Domain 2: soft skills

The majority of men and women preferred a doctor that takes the patient seriously, listens, informs on emotional needs, shows understanding and informs on wellbeing (i.e. asks how things are going). For the last two options the preference rates significantly differed between women and men. Showing understanding was preferred by 100% of women vs. 80% of men (*P* = 0.004). Informing on wellbeing was preferred by 100% of women vs. 72% of men (*P =* ≤0.000). Couples had most discrepant preferences towards counselling from a specialized nurse (level of intra-couple discrepancy 17%; preferred by 52% of both women and men) and counselling from a psychologist (level of intra-couple discrepancy 17%; preferred by 24% of women and 13% of men).

#### Domain 3: other types of supportive care

Options being preferred by the majority of women were: support from friends, support from family, more involvement of the male partner at the outpatient clinic (i.e. the doctor actively involves the male partner during consultations and in supportive care) and to talk to someone after a new miscarriage. The proportion of men that expressed a need for support from friends was significantly lower (48% vs. 74%, *P* = 0.017). None of the options in this domain were requested by ≥60% of men. More involvement of the male partner at the outpatient clinic was preferred by 70% of the women, compared to 37% of the men (*P* = 0.007). Sixty-one percent of women would like to talk to someone after experiencing another miscarriage, compared to 43% of men. The highest levels of intra-couple discrepancy were observed for need for support from peers (28%), followed by relaxation exercises (24%), yoga (24%) and talking to someone after a new miscarriage (22%).

Overall, the options for supportive care that were rejected by the majority of both women and men were bereavement therapy, listening to relaxation tapes, counselling from a social worker, counselling from a psychologist, alternative medication and hospital admission at the same gestational age as earlier miscarriages occurred. Alternative therapy (such as acupuncture or reflexology), relation exercises and yoga were not considered necessary by the majority of men. Mean levels of intra-couple discrepancy were 14% for Domain 1 (Medical supportive care), 9% for Domain 2 (Soft skills) and 17% for Domain 3 (Other types of supportive care).

## Discussion

This is the first study that quantified preferences for supportive care of both men and women affected by RPL and explored the existence of different needs within couples. Overall, men expressed a significantly lower need for supportive care compared to women. Regarding medical supportive care, preferences of both genders were largely similar and in line with the previous study in women by Musters et al. [13]. For the other domains of supportive care, several between-gender differences were observed.

Although the majority of both men and women preferred a doctor that takes the patient seriously, listens, informs on emotional needs, informs on wellbeing and shows understanding, a significantly smaller proportion of men appreciated the last two options (differences of 28 and 22% compared to women, respectively). In addition, the majority of women expressed a need for support from family, friends and peers; men preferred this less. This is in accordance with previous research showing that men are typically more hesitant to disclose their feelings after pregnancy loss [5, 18]. Although men do experience feelings of grief, stress and vulnerability, these emotions may be less manifested [19, 20]. Men are thought to employ different coping strategies compared to women, including ‘active avoidance’ and distractive behaviour, related to more frequently observed risk behaviours such as excessive alcohol consumption and smoking [5, 18]. Multiple studies showed that a significant part of men affected by pregnancy loss experienced little support from their social network and a reluctance to share their loss and feelings with them; their family and friends tend to direct their acknowledgement and support largely toward the female partner [5, 21, 22].

Also in hospital settings where support activities are profoundly targeted on or delivered by women, men have indicated that they feel excluded or marginalized from care compared to their partner [23]. In our study, remarkable gender differences were observed regarding the overall need for supportive care (mean grade 6.8 in men vs. 7.9 in women) and the need for more involvement of the male partner at the RPL outpatient clinic (desired by 37% of men and 70% of women). This seems in contrast with other studies indicating that male partners of RPL couples want to be more included [11, 14]. Multiple explanations may be underlying here. In some men’s responses, a social desirability bias may be present. Various studies on experiences following pregnancy loss showed that it is not uncommon for men to view their role as primarily being a ‘supporter’ to their female partner, leading to a barrier to seek support for themselves [18, 24–26]. Another possibility is that the approach at the clinic and the supportive care as it is currently being offered, do not completely meet the needs of men.

Furthermore, our results suggest that it is important to offer supportive care services to both partners individually. Although men and women may show similar preferences on group level, this does not automatically imply a high level of intra-couple agreement. For instance, while an equal percentage of the total groups of women and men (52%) preferred counselling from a specialized nurse during a next pregnancy, in almost one in five couples the partners had opposing opinions regarding this aspect (level of intra-couple discrepancy 17%). Moreover, in 28% of couples, one partner expressed a need for peer-support, while the other partner did not consider this necessary.

Previous research showed that patients with RPL want medical professionals to be aware of the psychological impact of RPL and believe they would benefit from psychological care [11, 14]. However, in the current study, the majority of both female and male participants rejected the options of being counselled by a psychologist or a social worker. Possibly, RPL patients consider it important that there is recognition of the psychological aspect of their losses by their healthcare providers, but they are not inclined to seek specialised psychological care. This may have to do with unfamiliarity with these types of care or perceived stigma and barriers to seek care from a mental health professional. Notably, preference rates for counselling from a specialised nurse were considerably higher.

The major strength of this study is that it is the first that quantified the need for different aspects of supportive care of both men and women affected by RPL. In a recent exploratory study in 13 couples with RPL, both members of the couples were interviewed simultaneously on their need for treatment, support and follow-up [11]. This likely resulted in each partner influencing the other’s perspectives, which was also recognized as a limitation by the authors themselves. In our study, the questionnaires returned by both members of each couple were carefully compared and no obvious overlap in their responses was present. This makes it credible that the questionnaires were completed independently of one another (as requested), although we cannot entirely rule out the possibility of some couples having discussed their responses. Moreover, it should be mentioned that responses of two partners will never be entirely independent, as they form a couple and they share the same experience. The study has several limitations. First, it is a single centre study and although the sample is representative for our RPL clinic, differences with RPL couples elsewhere may exist, for instance in terms of education level, being relatively high in our population. Likewise, services being offered in our RPL clinic may differ from other settings. Furthermore, the panel of supportive care options evaluated in this study was based on previous research restricted to women. It may be that some men desire other possibilities for supportive care, not being covered in this study.

It should be considered to develop supportive care programs for RPL specifically aimed at men, as supportive care in its current form may not entirely suit their needs. In a previous qualitative study, men affected by (single) pregnancy loss expressed a desire for an informal discussion with another man with the same experience. In a hospital setting, they suggested the option of a male support worker. Such possibilities may be further explored for men affected by RPL, for instance using focus group discussions, as mentioned in the study protocol of the currently ongoing study of Williams et al. [27].

## Conclusions

Our study shows the existence of different preferences for supportive care of men and women affected by RPL. It is important that health care providers are aware of this and take a tailored approach. We recommend to actively involve both partners, ask them about their personal preferences and discuss the most suitable approach that best fits the needs of both partners. It can be emphasized that some supportive care services may be chosen by one of the partners only. In addition, development of male-oriented supportive care programs should be explored.

## Supplementary Information

**Additional file 1.**

**Additional file 2: Supplementary Table 1.** Options for supportive care in a next pregnancy preferred by the majority (≥60%) of women and/or men.

## Data Availability

The datasets generated and/or analysed during the current study are available from the corresponding author (NF) on reasonable request. The data are not publicly available as they contain information that could compromise research participant privacy/consent.

## Abbreviations

APS: Antiphospholipid syndrome
IUI: Intrauterine insemination
IVF: In vitro fertilization
RPL: Recurrent pregnancy loss
SD: Standard deviation
TPO: Thyroid peroxidase
TSH: Thyroid-stimulating hormone
