# Supplementary material for: Exploring gender differences among couples with unexplained recurrent pregnancy loss regarding preferences for supportive care
Source: BMC Pregnancy Childbirth. 2021 Nov 30;21:796. doi: 10.1186/s12884-021-04277-4 (PMC8630871; doi:10.1186/s12884-021-04277-4)
Supplement: Supplementary file 1 — Additional file 1. [file 12884_2021_4277_MOESM1_ESM.pdf]

Supplementary material

Exploring gender differences in supportive care preferences of couples with recurrent pregnancy loss

Authors: NA du Fossé[a]*, EELO Lashley[a], TT Treurniet[a], JMM van Lith[a], S le Cessie[b,c], H Boosman[d], MLP van der Hoorn[a]

[a] Department of Gynecology and Obstetrics, Leiden University Medical Center, 2333 ZA Leiden, the Netherlands
[b] Department of Clinical Epidemiology, Leiden University Medical Center, 2333 ZA Leiden, the Netherlands
[c] Department of Biomedical Data Sciences, Leiden University Medical Center, 2333 ZA Leiden, the Netherlands
[d] Department of Quality and Patient Safety, Leiden University Medical Center, 2333 ZA Leiden, the Netherlands

Questionnaire

To be filled in by female partner (please without discussing with your partner)

1.1: General data

1. What is your date of birth (dd/mm/yyyy)?
   _ _ / _ _ / _ _

2. What is the country of birth of you and your parents?

	you	your father	your mother
the Netherlands	☐	☐	☐
other, namely	………………….	………………….	………………….

3. What is your highest educational qualification?
   - ☐ No degree
   - ☐ Intermediate vocational education
   - ☐ Higher general secondary education
   - ☐ Pre-university secondary education
   - ☐ University
   - ☐ other, namely…….

4. What is you religion?
   - ☐ Chrisitanity
   - ☐ Islam
   - ☐ Buddhism
   - ☐ Hinduism
   - ☐ Atheism / non-believing
   - ☐ Other, namely…….

5. What is your occupation?

   ……………………………………………………………………………………………………

6. Did you visit other hospitals for investigations or treatment regarding recurrent miscarriages?

   ☐ yes, in the Netherlands: ……………………………………………………………….

   ☐ yes, in other countries: ……………………………………………………

   ☐ no

1.2: Preferences for supportive care

The following questions are about your preferences for supportive care during a potential next pregnancy.

During a next pregnancy I would feel supported if I could make a plan with my doctor for the first 12 weeks of pregnancy

No, I do not prefer this ☐ ☐ ☐ ☐ ☐ Yes, I prefer this

During a next pregnancy I would feel supported if I would receive advice regarding nutrition

No, I do not prefer this ☐ ☐ ☐ ☐ ☐ Yes, I prefer this

During a next pregnancy I would feel supported if I would receive advice regarding lifestyle (what to do and what not to do)

No, I do not prefer this ☐ ☐ ☐ ☐ ☐ Yes, I prefer this

During a next pregnancy I would feel supported if I would receive an ultrasound

directly after a positive pregnancy test

No, I do not prefer this ☐ ☐ ☐ ☐ ☐ Yes, I prefer this

once a week

No, I do not prefer this ☐ ☐ ☐ ☐ ☐ Yes, I prefer this

once every two weeks

No, I do not prefer this ☐ ☐ ☐ ☐ ☐ Yes, I prefer this

during symptoms

No, I do not prefer this ☐ ☐ ☐ ☐ ☐ Yes, I prefer this

During a next pregnancy I would feel supported if I

would receive pregnancy hormone (βHCG) monitoring once before 1st ultrasound

No, I do not prefer this ☐ ☐ ☐ ☐ ☐ Yes, I prefer this

would receive pregnancy hormone (βHCG) monitoring more than one time before 1st ultrasound

No, I do not prefer this ☐ ☐ ☐ ☐ ☐ Yes, I prefer this

would be admitted to a hospital ward at the same gestational age as previous miscarriage

No, I do not prefer this ☐ ☐ ☐ ☐ ☐ Yes, I prefer this

would receive medication

No, I do not prefer this ☐ ☐ ☐ ☐ ☐ Yes, I prefer this

would receive medication, only if it has been proven safe for my pregnancy

No, I do not prefer this ☐ ☐ ☐ ☐ ☐ Yes, I prefer this

During a next pregnancy I would feel supported if I have

one doctor

No, I do not prefer this ☐ ☐ ☐ ☐ ☐ Yes, I prefer this

two doctors

No, I do not prefer this ☐ ☐ ☐ ☐ ☐ Yes, I prefer this

more than two doctors

No, I do not prefer this ☐ ☐ ☐ ☐ ☐ Yes, I prefer this

During a next pregnancy I would feel supported if my doctor(s):

has knowledge of my obstetric history

No, I do not prefer this ☐ ☐ ☐ ☐ ☐ Yes, I prefer this

has knowledge of my home situation

No, I do not prefer this ☐ ☐ ☐ ☐ ☐ Yes, I prefer this

shows understanding

No, I do not prefer this ☐ ☐ ☐ ☐ ☐ Yes, I prefer this

informs on emotional needs

No, I do not prefer this ☐ ☐ ☐ ☐ ☐ Yes, I prefer this

takes me seriously

No, I do not prefer this ☐ ☐ ☐ ☐ ☐ Yes, I prefer this

listens to me

No, I do not prefer this ☐ ☐ ☐ ☐ ☐ Yes, I prefer this

informs on how I am doing

No, I do not prefer this ☐ ☐ ☐ ☐ ☐ Yes, I prefer this

During a subsequent I would feel supported if I would receive support from

A specialized nurse

No, I do not prefer this ☐ ☐ ☐ ☐ ☐ Yes, I prefer this

A social worker

No, I do not prefer this ☐ ☐ ☐ ☐ ☐ Yes, I prefer this

A psychologist

No, I do not prefer this ☐ ☐ ☐ ☐ ☐ Yes, I prefer this

Someone else, namely …………………………………………………..

No, I do not prefer this ☐ ☐ ☐ ☐ ☐ Yes, I prefer this

During a next pregnancy, I'm looking for support from

My family

No, I do not prefer this ☐ ☐ ☐ ☐ ☐ Yes, I prefer this

My friends

No, I do not prefer this ☐ ☐ ☐ ☐ ☐ Yes, I prefer this

People in the same situation

No, I do not prefer this ☐ ☐ ☐ ☐ ☐ Yes, I prefer this

During a next pregnancy I would be able to relax and feel supported if

I would listen to relaxation tapes

No, I do not prefer this ☐ ☐ ☐ ☐ ☐ Yes, I prefer this

I would do relaxation exercises

No, I do not prefer this ☐ ☐ ☐ ☐ ☐ Yes, I prefer this

I would do yoga exercises

No, I do not prefer this ☐ ☐ ☐ ☐ ☐ Yes, I prefer this

I would participate in a bereavement therapy/course

No, I do not prefer this ☐ ☐ ☐ ☐ ☐ Yes, I prefer this

During a next pregnancy I would feel supported if I would receive information about my pregnancy from

my doctor(s)

No, I do not prefer this ☐ ☐ ☐ ☐ ☐ Yes, I prefer this

the internet

No, I do not prefer this ☐ ☐ ☐ ☐ ☐ Yes, I prefer this

people in the same situation

No, I do not prefer this ☐ ☐ ☐ ☐ ☐ Yes, I prefer this

During a next pregnancy I would feel supported if my partner would be more involved

No, I do not prefer this ☐ ☐ ☐ ☐ ☐ Yes, I prefer this

During a next pregnancy I would feel supported if I

Would receive alternative medicine (homeopathic or Chinese medication, etc)

No, I do not prefer this ☐ ☐ ☐ ☐ ☐ Yes, I prefer this

Would receive alternative therapies (acupuncture, reflexology etc)

No, I do not prefer this ☐ ☐ ☐ ☐ ☐ Yes, I prefer this

During a next pregnancy I would feel supported if I

could be waiting in a waiting room without visibly pregnant woman

No, I do not prefer this ☐ ☐ ☐ ☐ ☐ Yes, I prefer this

If my next pregnancy was to end in a miscarriage I would feel supported if I could talk to someone

No, I do not prefer this ☐ ☐ ☐ ☐ ☐ Yes, I prefer this

What is your need for supportive care during your next pregnancy?

(circle your response: 1 = very little need, 10 = very much need)

1 2 3 4 5 6 7 8 9 10

Do you have any comments or additions?

……

……

……

……

……

……

……

……

……

……

Questionnaire

To be filled in by male partner (please do without discussing with your partner)

1.1: General data

1. What is your date of birth (dd/mm/yyyy)?
   _ _ / _ _ / _ _

2. What is the country of birth of you and your parents?

	you	your father	your mother
the Netherlands	☐	☐	☐
other, namely	………………….	………………….	………………….

3. What is your highest educational qualification?
   - ☐ No degree
   - ☐ Intermediate vocational education
   - ☐ Higher general secondary education
   - ☐ Pre-university secondary education
   - ☐ University
   - ☐ other, namely…….

4. What is you religion?
   - ☐ Chrisitanity
   - ☐ Islam
   - ☐ Buddhism
   - ☐ Hinduism
   - ☐ Atheism / non-believing
   - ☐ Other, namely…….

5. What is your occupation?

   …………………………………………………………………………………………………

1.2: Preferences for supportive care

The following questions are about your preferences for supportive care during a potential next pregnancy.

During a next pregnancy I would feel supported if we could make a plan with my doctor for the first 12 weeks of pregnancy

No, I do not prefer this ☐ ☐ ☐ ☐ ☐ Yes, I prefer this

During a next pregnancy I would feel supported if my partner would receive advice regarding nutrition

No, I do not prefer this ☐ ☐ ☐ ☐ ☐ Yes, I prefer this

During a next pregnancy I would feel supported if my partner would receive advice regarding lifestyle (what to do and what not to do)

No, I do not prefer this ☐ ☐ ☐ ☐ ☐ Yes, I prefer this

During a next pregnancy I would feel supported if my partner would receive an ultrasound

directly after a positive pregnancy test

No, I do not prefer this ☐ ☐ ☐ ☐ ☐ Yes, I prefer this

once a week

No, I do not prefer this ☐ ☐ ☐ ☐ ☐ Yes, I prefer this

once every two weeks

No, I do not prefer this ☐ ☐ ☐ ☐ ☐ Yes, I prefer this

during symptoms

No, I do not prefer this ☐ ☐ ☐ ☐ ☐ Yes, I prefer this

During a next pregnancy I would feel supported if my partner

would receive pregnancy hormone (βHCG) monitoring once before 1st ultrasound

No, I do not prefer this ☐ ☐ ☐ ☐ ☐ Yes, I prefer this

would receive pregnancy hormone (βHCG) monitoring more than one time before 1st ultrasound

No, I do not prefer this ☐ ☐ ☐ ☐ ☐ Yes, I prefer this

would be admitted to a hospital ward at the same gestational age as previous miscarriage

No, I do not prefer this ☐ ☐ ☐ ☐ ☐ Yes, I prefer this

would receive medication

No, I do not prefer this ☐ ☐ ☐ ☐ ☐ Yes, I prefer this

would receive medication, only if it has been proven safe for my pregnancy

No, I do not prefer this ☐ ☐ ☐ ☐ ☐ Yes, I prefer this

During a next pregnancy I would feel supported if we have

one doctor

No, I do not prefer this ☐ ☐ ☐ ☐ ☐ Yes, I prefer this

two doctors

No, I do not prefer this ☐ ☐ ☐ ☐ ☐ Yes, I prefer this

more than two doctors

No, I do not prefer this ☐ ☐ ☐ ☐ ☐ Yes, I prefer this

During a next pregnancy I would feel supported if my doctor(s):

has knowledge of my partners obstetric history

No, I do not prefer this ☐ ☐ ☐ ☐ ☐ Yes, I prefer this

has knowledge of my home situation

No, I do not prefer this ☐ ☐ ☐ ☐ ☐ Yes, I prefer this

shows understanding

No, I do not prefer this ☐ ☐ ☐ ☐ ☐ Yes, I prefer this

informs on emotional needs

No, I do not prefer this ☐ ☐ ☐ ☐ ☐ Yes, I prefer this

takes me seriously

No, I do not prefer this ☐ ☐ ☐ ☐ ☐ Yes, I prefer this

listens to me

No, I do not prefer this ☐ ☐ ☐ ☐ ☐ Yes, I prefer this

informs on how I am doing

No, I do not prefer this ☐ ☐ ☐ ☐ ☐ Yes, I prefer this

During a subsequent I would feel supported if I would receive support from

A specialized nurse

No, I do not prefer this ☐ ☐ ☐ ☐ ☐ Yes, I prefer this

A social worker

No, I do not prefer this ☐ ☐ ☐ ☐ ☐ Yes, I prefer this

A psychologist

No, I do not prefer this ☐ ☐ ☐ ☐ ☐ Yes, I prefer this

Someone else, namely ……………………………………………….

No, I do not prefer this ☐ ☐ ☐ ☐ ☐ Yes, I prefer this

During a next pregnancy, I'm looking for support from

My family

No, I do not prefer this ☐ ☐ ☐ ☐ ☐ Yes, I prefer this

My friends

No, I do not prefer this ☐ ☐ ☐ ☐ ☐ Yes, I prefer this

People in the same situation

No, I do not prefer this ☐ ☐ ☐ ☐ ☐ Yes, I prefer this

During a next pregnancy I would be able to relax and feel supported if

I would listen to relaxation tapes

No, I do not prefer this ☐ ☐ ☐ ☐ ☐ Yes, I prefer this

I would do relaxation exercises

No, I do not prefer this ☐ ☐ ☐ ☐ ☐ Yes, I prefer this

I would do yoga exercises

No, I do not prefer this ☐ ☐ ☐ ☐ ☐ Yes, I prefer this

I would participate in a bereavement therapy/course

No, I do not prefer this ☐ ☐ ☐ ☐ ☐ Yes, I prefer this

During a next pregnancy I would feel supported if I would receive information about the pregnancy from

my doctor(s)

No, I do not prefer this ☐ ☐ ☐ ☐ ☐ Yes, I prefer this

the internet

No, I do not prefer this ☐ ☐ ☐ ☐ ☐ Yes, I prefer this

people in the same situation

No, I do not prefer this ☐ ☐ ☐ ☐ ☐ Yes, I prefer this

During a next pregnancy I would feel supported if I would be more involved

No, I do not prefer this ☐ ☐ ☐ ☐ ☐ Yes, I prefer this

During a next pregnancy I would feel supported if my partner

Would receive alternative medicine (homeopathic or Chinese medication, etc)

No, I do not prefer this ☐ ☐ ☐ ☐ ☐ Yes, I prefer this

Would receive alternative therapies (acupuncture, reflexology etc)

No, I do not prefer this ☐ ☐ ☐ ☐ ☐ Yes, I prefer this

During a next pregnancy I would feel supported if I

could be waiting in a waiting room WITHOUT visibly pregnant woman

No, I do not prefer this ☐ ☐ ☐ ☐ ☐ Yes, I prefer this

If the next pregnancy was to end in a miscarriage I would feel supported if I could talk to someone

No, I do not prefer this ☐ ☐ ☐ ☐ ☐ Yes, I prefer this

What is your need for supportive care during a next pregnancy?

(circle your response: 1 = very little need, 10 = very much need)

1 2 3 4 5 6 7 8 9 10

Do you have any comments or additions?

………

………

………

………

………

………

………

………

………

………
