# Supplementary material for: Exploring gender differences among couples with unexplained recurrent pregnancy loss regarding preferences for supportive care
Source: BMC Pregnancy Childbirth. 2021 Nov 30;21:796. doi: 10.1186/s12884-021-04277-4 (PMC8630871; doi:10.1186/s12884-021-04277-4)
Supplement: Supplementary file 2 — Additional file 2: Supplementary Table 1. Options for supportive care in a next pregnancy preferred by the majority (≥60%) of women and/or men. [file 12884_2021_4277_MOESM2_ESM.docx]

**Supplementary material**

**Exploring gender differences in supportive care preferences of couples with recurrent pregnancy loss**

**Authors:** NA du Fossé^a^*, EELO Lashley^a^, TT Treurniet^a^, JMM van Lith^a^, S le Cessie^b,c^, H Boosman^d^, MLP van der Hoorn^a^

^a^ Department of Gynecology and Obstetrics, Leiden University Medical Center, 2333 ZA Leiden, the Netherlands
^b^ Department of Clinical Epidemiology, Leiden University Medical Center, 2333 ZA Leiden, the Netherlands
^c^ Department of Biomedical Data Sciences, Leiden University Medical Center, 2333 ZA Leiden, the Netherlands
^d^ Department of Quality and Patient Safety, Leiden University Medical Center, 2333 ZA Leiden, the Netherlands

**Supplementary Table 1**

**Options for supportive care in a next pregnancy preferred by the majority (≥60%) of women and/or men**

|  | Women  *n* = 46 | | | Men  *n* = 46 | | |
| --- | --- | --- | --- | --- | --- | --- |
| Medical supportive care *n* (%) | **No need** | **Neutral** | **Prefer** | **No need** | **Neutral** | **Prefer** |
| Plan for first trimester  The same doctor  Doctor has knowledge of obstetric history  Ultrasound directly after  positive pregnancy test  Ultrasound during symptoms  Ultrasound once a week  Information from doctor  Medication proven safe for pregnancy | 5 (11)  5 (11)  0 (0)  13 (28)  2 (4)  11 (24)  1 (2)  6 (13) | 4 (9)  7 (15)  0 (0)  3 (7)  3 (7)  3 (7)  2 (4)  6 (13) | 37 (80)  33 (72)  46 (100)  30 (65)  40 (87)  30 (65)  43 (93)  34 (74) | 3 (7)  5 (11)  0 (0)  6 (13)  1 (2)  11 (24)  0 (0)  2 (4) | 7 (15)  8 (17)  2 (4)  8 (17)  7 (15)  8 (17)  8 (17)  9 (20) | 36 (78)  32 (70)  44 (96)  32 (70)  37 (80)  26 (57)  38 (83)  34 (74) |
| Soft skills *n* (%) |  |  |  |  |  |  |
| *Doctor:*  Takes you seriously  Listens to you  Shows understanding  Informs on wellbeing  Informs on emotional needs | 0 (0)  0 (0)  0 (0)  0 (0)  1 (2) | 1 (2)  0 (0)  0 (0)  0 (0)  5 (11) | 44 (96)  46 (100)  46 (100)  46 (100)  40 (87) | 0 (0)  0 (0)  0 (0)  3 (7)  4 (9) | 2 (4)  4 (9)  9 (20)  10 (22)  9 (20) | 44 (96)  42 (91)  37 (80)  33 (72)  33 (72) |
| Other types of supportive care *n* (%) |  |  |  |  |  |  |
| *Support* *from:*  Friends  Family  More involvement of male partner at outpatient clinic  Talk to someone after miscarriage | 4 (9)  4 (9)  3 (7)  8(17) | 8 (17)  7 (15)  11 (24)  9 (20) | 34 (74)  35 (76)  32 (70)  28 (61) | 6 (13)  4 (9)  2 (4)  14 ( 30) | 18 (39)  16 (35)  26 (57)  11 (24) | 22 (48)  26 (57)  17 (37)  20 (43) |
